# Association of RSV-A ON1 genotype with Increased Pediatric Acute Lower Respiratory Tract Infection in Vietnam

**DOI:** 10.1038/srep27856

**Published:** 2016-06-16

**Authors:** Keisuke Yoshihara, Minh Nhat Le, Michiko Okamoto, Anita Carolle Akpeedje Wadagni, Hien Anh Nguyen, Michiko Toizumi, Enga Pham, Motoi Suzuki, Ai Thi Thuy Nguyen, Hitoshi Oshitani, Koya Ariyoshi, Hiroyuki Moriuchi, Masahiro Hashizume, Duc Anh Dang, Lay-Myint Yoshida

**Affiliations:** 1 Department of Pediatric Infectious Diseases, Institute of Tropical Medicine, Nagasaki University, Nagasaki, 852-8523, Japan; 2 Leading Program, Graduate School of Biomedical Science, Nagasaki University, Nagasaki, 852-8523, Japan; 3 National Institute of Hygiene and Epidemiology, Hanoi, Vietnam; 4 Department of Virology, Tohoku University Graduate School of Medicine, Sendai, 980-8575, Japan; 5 Graduate School of Biomedical Sciences, Nagasaki University, Nagasaki, 852-8523, Japan; 6 Khanh Hoa General Hospital, Nha Trang, Vietnam; 7 Department of Clinical Medicine, Institute of Tropical Medicine, Nagasaki University, Nagasaki, 852-8523, Japan; 8 Department of Pediatrics, Nagasaki University Hospital, Nagasaki, 852-8102, Japan

## Abstract

Since the initial discovery of RSV-A ON1 in Canada in 2010, ON1 has been reported worldwide, yet information regarding its clinical impact and severity has been controversial. To investigate the clinical relevance of RSV-A ON1,acute respiratory infection (ARI) cases enrolled to our population-based prospective pediatric ARI surveillance at Khanh Hoa General Hospital, Central Vietnam from January 2010 through December 2012 were studied. Clinical-epidemiological information and nasopharyngeal samples were collected. Multiplex PCR assays were performed for screening 13 respiratory viruses. RSV-positive samples were further tested for subgroups (A/B) and genotypes information by sequencing the G-glycoprotein 2nd hypervariable region. Statistical analysis was performed to evaluate the clinical-epidemiological characteristics of RSV-A ON1. A total of 1854 ARI cases were enrolled and 426 (23.0%) of them were RSV-positive. During the study period, RSV-A and B had been co-circulating. NA1 was the predominant RSV-A genotype until the appearance of ON1 in 2012. RSV-related ARI hospitalization incidence significantly increased after the emergence of ON1. Moreover, multivariate analysis revealed that risk of lower respiratory tract infection was 2.26 (95% CI: 1.37–3.72) times, and radiologically-confirmed pneumonia was 1.98 (95% CI: 1.01–3.87) times greater in ON1 compared to NA1 cases. Our result suggested that ON1 ARI cases were clinically more severe than NA1.

Human Respiratory Syncytial Virus (RSV) is a pneumovirus under the family of paramyxoviridae with a negative-sense single stranded RNA genome^1^. RSV is widely known as one of the most common respiratory viral pathogens for lower respiratory tract infection (LRTI) among infants and young children worldwide^2^. RSV-related Acute Respiratory Infection (ARI) morbidity causes huge public health concerns particularly among children less than 5 with more than 60 million LRTI episodes annually in developing nations^3^. Previously, our population-based ARI surveillance in Central Vietnam reported that RSV was one of the main viral pathogens among hospitalized ARI children less than 2 years of age^4^. In fact, virtually all the children are exposed to RSV at some point before 2 years of age^5^. Furthermore, recurrence of RSV infections is common and particularly cause life-threatening LRTI in children less than 6 months^6^. Younger age, lower socioeconomic status, shorter gestational age, prematurity, low birthweight, lack of breastfeeding and family smoking have been previously reviewed as risk-factors for developing severe RSV-related LRTIs^7^,^8^,^9^.

RSV is classified into two antigenically and genetically distinct subgroups (A/B)^10^. Each subgroup is further categorized into genotypes based on the nucleotide sequence variation within the 2nd hypervariable region of heavily glycosylated G-glycoprotein (G-protein). There are 12 genotypes for RSV-A (GA1-7, SAA1, NA1-2 and ON1-2) and 20 genotypes for RSV-B (GB1-4, SAB1-4, URU1-2 and BA1-10)^11^,^12^,^13^. Primary function of the G-protein is associated with viral attachment to the cell receptors and acts as an immunologic peptide that induces neutralizing antibody^14^,^15^,^16^. The relevance of distinct RSV subgroups with clinical manifestations has been controversial. For instance, the studies from the U.S. and Argentina previously demonstrated that RSV-A was most likely associated with clinical severity^17^,^18^; however, a study from Brazil presented the opposite result^19^, while the other studies showed no significant difference^20^,^21^,^22^. Furthermore, the studies from Canada and the U.S. implicated that RSV-A GA2 or RSV-B GB3 genotype may be linked to clinical severity^20^,^23^. To gain a better understanding of the association of RSV subgroup and genotype with clinical outcome, it is essential to further expand RSV molecular epidemiological surveillance worldwide.

In 2010, ON1 genotype, a new variant of RSV-A, was initially detected in Ontario, Canada by Eshaghi *et al.*^24^. As a unique genetic characteristic, ON1 possesses 72-nucleotide tandem duplication within the G-protein 2nd hypervariable region. Since the first discovery of ON1 in Canada^24^, it has been reported in countries around the globe^11^,^25^,^26^,^27^,^28^,^29^,^30^,^31^, including South East Asian countries such as Philippines, Malaysia and Thailand^32^,^33^,^34^. Furthermore, the reports from Cyprus, Germany, Italy, Kenya and Philippine indicated that the emergence of ON1 has rapidly replaced the previously predominant NA1 genotype^29^,^30^,^31^,^34^,^35^,^36^. Although the clinical aspects of ON1 genotype were investigated in the previous reports from Cyprus, Germany and Italy^29^,^30^,^31^, the clinical relevance and pathogenicity of newly emerged ON1 genotype remain unclear.

There is still limited information regarding the molecular and clinical epidemiological characteristics of RSV particularly in South East Asian nations including Vietnam. Therefore in this study, we investigated the annual incidence of RSV-related pediatric ARI hospitalization, circulation dynamics of RSV subgroups (A/B) and genotypes, and the clinical significance of RSV-A ON1 genotype among the hospitalized pediatric ARI cases in Central Vietnam.

## Results

### Enrolled pediatric ARI cases and hospitalization incidence

A total of 1854 hospitalized acute respiratory infection (ARI) cases were enrolled into our population-based pediatric ARI surveillance at Khanh Hoa General Hospital (KHGH) during the three years study period. Chest X-ray result were available for 1796 (96.9%) of enrolled ARI cases. Annual numbers of pediatric ARI cases enrolled were 542 in 2010, 513 in 2011 and 799 in 2012 (Table 1). Based on the 2010 population census data, the pediatric ARI hospitalization incidence rates were 3976.2 cases per 100,000 children under 5 per year (95% CI: 3654.4–4317.9) in 2010, 3763.5 (95% CI: 3450.2–4096.7) in 2011 and 5861.6 (95% CI: 5473.1–6269.2) in 2012 respectively. Result of the respiratory virus screening by multiplex PCR assays revealed that viruses were detected in 66.2% of the enrolled ARI cases, in which RSV (n = 426, 23.0%), Influenza (overall, 12.0%: type-A, 7.5% and type-B, 4.5%) and Rhinovirus (25.1%) were the leading respiratory viruses detected (data not shown).

### Incidence and seasonality of RSV ARI cases

**Figure 1:**
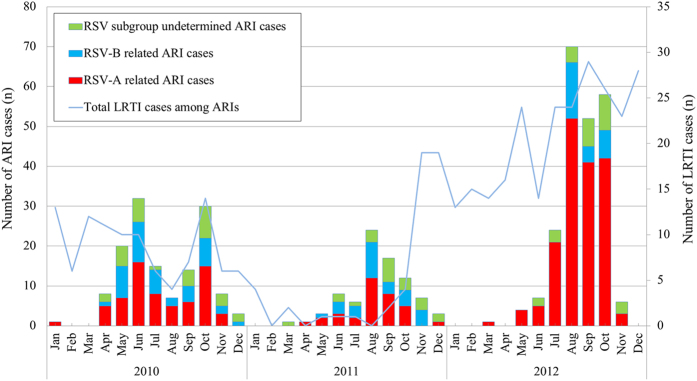
Seasonal trend of RSV related pediatric ARI hospitalizations in Nha Trang, Khanh Hoa province during January 2010–December 2012. Each box corresponds to the cumulative pediatric ARI cases in each month during the study period from January 2010 to December 2012. RED-filled boxes correspond to the RSV subgroup-A ARI cases, and BLUE-filled boxes are for the RSV subgroup-B ARI cases. RSV subgroup unclassified ARI cases were added on top of the BLUE-filled boxes as GREEN-filled boxes. Lower respiratory tract infection (LRTI) in each month was indicated as the BLUE solid line. *ARI is abbreviation for “Acute Respiratory Infection”. *LRTI is abbreviation for “lower respiratory tract infection”.

RSV-related hospitalized ARI cases in Central Vietnam presented a clear seasonal circulation pattern with peaks in hot and dry seasons, which extended from July to September (Fig. 1). The RSV-related ARI hospitalization incidence rates varied yearly: 990.4 (95% CI: 831.0–1171.2) in 2010, 586.9 (95% CI: 465.6–729.9) in 2011 and 1547.9 (95% CI: 1347.4–1769.5) in 2012 season respectively. The highest RSV-related ARI hospitalization incidence recorded in 2012 corresponded to the highest LRTI incidence in the same season, accounting for 1804.7 (95% CI: 1587.9–2042.4) (Table 1).

### Demographic and clinical characteristics of RSV and Non-RSV ARI cases

Demographic characteristics of the RSV ARI cases (n = 426) were compared with non-RSV ARI cases (n = 1428) (Table 2). Both RSV and non-RSV ARI cases were more common among males (59.6%, RSV vs 56.0%, non-RSV, *p* = 0.179). RSV-positive ARI cases were significantly younger (median age in month) (12, RSV vs 17, non-RSV, *p* < 0.001) and more commonly seen in the first year of life (51.2%, RSV vs 37.0%, non-RSV cases, *p* < 0.001). Daycare attendance was less common among the RSV-positive ARI cases (32.2%, RSV vs 38.7%, non-RSV, *p* = 0.015), and antibiotic usage prior to hospital admission was less frequent among the RSV-positive ARI cases (39.7% vs 44.3%, *p* = 0.042). Furthermore, the presence of underlying medical condition was commonly seen in non-RSV ARI cases (31.2%, RSV vs 37.9%, non-RSV, *p* = 0.012).

In terms of the clinical presentation comparison, wheezing (52.8%, RSV vs 45.9%, non-RSV, *p* = 0.012), tachypnea (31.0%, RSV vs 21.1%, non-RSV, *p* < 0.001), crackle (21.6%, RSV vs 16.3%, non-RSV, *p* = 0.012) and chest-wall indrawing (9.9%, RSV vs 6.8%, non-RSV, *p* = 0.035) were significantly more common among the RSV-positive ARI cases (Table 2). Furthermore, the association of RSV-positive ARI cases with greater clinical severity remained significant even after controlling for the demographic confounding factors in the multivariate regression analysis (Table S1).

### Prevalence and incidence of RSV subgroup A and B ARI cases

RSV subgrouping and genotyping were performed for all the RSV-positive confirmed ARI samples (n = 426). We were able to classify the subgroup (A/B) in 346 RSV-positive ARI cases: 253 RSV-A and 77 RSV-B respectively (Table 1). We found that 16 samples were co-infected with both RSV subgroup A and B. Throughout the study period from January 2010 to December 2012, RSV-A was the major subgroup identified. RSV-A related ARI hospitalization incidences rates (per 100,000) were 462.2 (in 2010), 234.8 (in 2011) and 1159.1 (in 2012) respectively. The RSV-B ARI hospitalization rates were 278.8 (in 2010), 183.4 (in 2011) and 102.7 (in 2012) respectively. Notably, the total number and proportion of RSV-A related ARI hospitalization dramatically increased in 2012 season (Fig. 1), which was statistically significant compared to the previous two seasons (*p* < 0.001) (Table 3).

### Prevalence of RSV subgroup A and B genotypes

Genotyping was performed for all the RSV subgroup (A/B) confirmed ARI samples by sequencing and phylogenetic analysis of the G-protein 2nd hypervariable region (Figs S1 and 2). All the RSV-A confirmed ARI samples from both 2010 and 2011 seasons were NA1 genotype, while in 2012, RSV-A ON1 emerged and immediately became the predominant RSV-A genotype. On the other hand, the proportion of NA1 among the RSV subgroup A confirmed ARI samples decreased to 22%. Overall, RSV-A phylogenetic tree presented distinct genetic clustering of the ARI hospitalization cases in 2010–2011 seasons from 2012 season. RSV-A ARI cases from 2010–2011 seasons were bundled into NA1 whereas RSV-A from 2012 season formed distinct cluster within ON1 genotype (Fig. S1). In fact, NA1 genotype was further divided into genetically distinct clades (1 or 2). Eight RSV-A confirmed ARI samples were not able to be categorized into genotype (Table 3).

With respect to the RSV-B genotype circulation pattern, the phylogenetic tree revealed that BA9, BA10 and BA-C had been circulating during the three years study period (Fig. S2). Overall, there was no noticeable genotype shift in RSV subgroup B during the study period (Table 3).

### Demographic and clinical characteristics of RSV-A ON1 and NA1 genotype

To investigate the clinical impact of RSV-A ON1 genotype, we compared the demographic and clinical characteristics of RSV-A ON1 with NA1 genotype (Table 4). A total of 123 RSV-A ON1 and 138 NA1 genotype confirmed ARI cases were included in the statistical analysis.

The overall demographic characteristics were similar between ON1 and NA1 ARI cases. ON1 ARI cases was younger (median age in month) (11, ON1 vs 13, NA1, *p* = 0.020) with slightly more NA1 ARI cases in the older age groups (Table 4). Daycare attendance was significantly higher among the NA1 ARI cases (24.4%, ON1 vs 39.1%, NA1, *p* = 0.011). Furthermore, the ON1 ARI cases were more frequently associated with underlying medical condition (43.1%, ON1 vs 21.7%, NA1, *p* < 0.001).

Regarding the respiratory clinical signs and symptoms, occurrence of respiratory sign and symptoms such as wheeze (81.3%, ON1 vs 33.3%, NA1, *p* < 0.001), tachypnea (50.4%, ON1 vs 25.4%, NA1, *p* < 0.001) and difficulty in breathing (18.7%, ON1 vs 6.5%, NA1, *p* = 0.004) were significantly more common among ON1 ARI cases (Table 4). In addition, ARI cases with LRTI (34.2%, ON1 vs 15.2%, NA1, *p* < 0.001) and radiologically-confirmed pneumonia (19.5%, ON1 vs 8.7%, NA1, *p* = 0.011) were more commonly seen in ON1 ARI cases. Furthermore, ON1 ARI cases tended to be admitted to the hospital significantly earlier, since they had a shorter mean period from disease onset to the hospital admission (in day) (1.7, ON1 vs 2.7, NA1, *p* < 0.001). On the other hand, mean duration of hospitalization (in day) between ON1 and NA1 ARI cases did not differ significantly (5.3, ON1 vs 5.1, NA1, *p* = 0.329).

### Relative risk of clinical features between RSV-A ON1 and NA1 genotype

The respiratory clinical signs and symptoms that presented significant difference in proportion between ON1 and NA1 ARI cases in Table 4 were further proceeded to the multivariate regression analysis. Multivariate analysis with log-binominal regression was performed to estimate the relative risks (RR) (Table 5). Sex, age, antibiotic-use, daycare attendance, viral co-infection and underlying medical condition were adjusted for estimating adjusted Relative Risk (Adj RR).

Result of the multivariate regression analysis illustrated the significant association of ON1 ARI cases with greater clinical severity even after controlling for the demographic confounding factors. For instance, ON1 ARI cases had greater risk of wheezing (Adj RR: 2.21 (95% CI: 1.72–2.86)), tachypnea (Adj RR: 1.83 (95% CI: 1.30–2.57)) and difficulty in breathing (Adj RR: 2.46 (95% CI: 1.12–5.39)). Furthermore, RSV-A ON1 had significantly greater risk for severe LRTI (Adj RR: 2.42 (95% CI: 1.12–5.25)) and radiologically-confirmed pneumonia (Adj RR: 1.97 (95% CI: 1.04–3.74)). On the other hand, relative risk for SpO2 (≦90%) and mild LRTI became no longer significant in the multivariate analysis: SpO2 (≦90%) (Adj RR: 3.18 (95% CI: 0.71–14.29)) and mild LRTI (Adj RR: 1.94 (95% CI: 0.90–4.19)) respectively.

## Discussion

Our study illustrated that RSV played a major clinical role among pediatric ARI cases in Central Vietnam. The result illustrated that RSV-related pediatric ARI hospitalization incidences were high during hot and dry season (July through September), timing of which was similar to previous findings from Cambodia, Thailand, Vietnam^4^,^37^,^38^. However correlation between climatic parameters (such as temperature and relative humidity) and RSV incidences is not clearly understood in tropical climate regions like Vietnam^39^. Future studies are required to clarify RSV seasonality in tropical countries.

The majority of RSV-related ARI cases were detected among children less than 2 years of age (Table 2), which was consistent with the previous finding^5^. According to the RSV subgroup specific circulation dynamics in our study site, RSV-A and B had been co-circulating during the three years study period from January 2010 to December 2012. RSV subgroup A was predominant throughout the study period, which was similar to previous studies in other South East Asian Countries^32^,^33^,^34^. In 2012 season, the RSV-A related ARI hospitalization incidence increased remarkably (1159.1 cases per 100,000) compared to the previous two seasons, which corresponded to the highest LRTI incidence recorded in the same season (1804.7 cases per 100,000) (Table 1).

Since the initial discovery of RSV-A ON1 genotype in Ontario, Canada in 2010^24^, ON1 have been reported in numbers of European and Asian nations primarily during 2010–2012 season^11^,^25^,^26^,^27^,^28^,^29^,^30^,^31^,^32^,^33^,^34^,^35^,^36^,^40^. This study is the first report regarding the emergence of ON1 in Vietnam. In our study site, NA1 was circulating as the only RSV-A genotype in 2010 and 2011 seasons (Table 3). However, ON1 emerged in mid-2012 and became the predominant genotype in 2012 season. The prevalence of ON1 among RSV-A reached nearly 73% in 2012 season whereas proportion of NA1 decreased down to about 23% from 100% in both 2010 and 2011 season (Table 3). ON1 genotype emerged and was detected as major genotype during 2011–2012 seasons in countries such as Cyprus, Italy, Germany and Philippine^29^,^30^,^31^,^34^,^35^,^36^. However, further studies are required to monitor whether ON1 persisted as the predominant genotype after its emergence in these countries. On the other hand, studies from Canada, China, Thailand and Malaysia reported its emergence but did not find ON1 genotype as the most prevalent type in their report^11^,^24^,^32^,^33^,^40^.

Regardless of the numbers of molecular epidemiological surveillances on RSV describing the emergence of ON1 genotype during the last couple of years, the clinical and pathological significance of RSV-A ON1 and its 72-nucleotide tandem duplication within the G-protein 2nd hypervariable region has not been clearly understood. Since our current pediatric ARI surveillance possessed a relatively large sample size in both RSV-A genotype ON1 (n = 123) and NA1 (n = 138) ARI cases, we were able to evaluated the demographic and clinical characteristics of ON1 ARI cases, in comparison with NA1 ARI cases (Table 4).

With respect to the demographic characteristics, ON1 ARI cases were seen in slightly younger (median age in month) (11, ON1 vs 13, NA1, *p* = 0.020), which may explain the lower prevalence of daycare attendance in the ON1 ARI cases (24.4%, ON1 vs 39.1%, NA1, *p* = 0.011). One study from Italy, Pierangeli *et al.* also found that ON1 cases were seen in slightly younger age group^30^, while other studies did not find significant differences^31^,^36^. The discrepancies in age distribution may have occurred due to differences in study design, method for case enrollment criteria, herd-immunity against RSV (subgroups and genotypes) and circulating RSV genotypes prior to ON1 emergence in each study.

With respect to the clinical characteristics, the multivariate analysis using log-binomial regression revealed that ON1 ARI cases were associated with increased risk of respiratory clinical signs/symptoms and severity compared to NA1 ARI cases. For instance, risk of wheezing was 2.21 (95% CI: 1.72–2.86) times, LRTI was 2.26 (95% CI: 1.37–3.72) times, and chest X-ray abnormality was 2.14 (95% CI: 1.13–4.04) times greater among ON1 ARI cases compared to NA1 ARI cases (Table 5). Furthermore, significantly shorter mean period from disease onset to the hospital admission (in day) was seen in ON1 ARI cases (1.7, ON1 vs 2.7, NA1, *p* < 0.001). Although the detailed biological mechanism has not been clearly understood, the G-protein 72-nucleotide tandem duplication of ON1 might have crucial biological role by enhancing the efficiency for viral attachment to the cell receptors or faster viral replication capacity during pathogenesis. Further studies are necessary to clarify the biological significance of the 72-nucleotide insertion in the G-protein.

In contrast to the our major finding of ON1’s association with clinical severity, other RSV surveillances from Cyprus, Germany and Italy did not find any remarkable clinical impact of ON1 genotype^29^,^31^,^36^,^41^. The clinical impact of a newly emerged virus may depend on the herd-immunity in the community, pre-circulating viruses and genotypes in respective study area. Recently, two studies have described the molecular evolutionary characteristics of globally circulating RSV-A NA1 and ON1 genotype^13^,^42^. Further genetic and antigenic analysis on the Central Vietnam RSV-A genotypes will give us insight into possible underlying mechanisms of the association between RSV ON1 and clinical severity.

As considerable limitations in the current study, we were not able to categorize RSV subgroups (A/B) or genotypes in about 20% of RSV confirmed ARI samples (Table 3). This may have been due to the fact that we used the RNA extracted directly from NP samples , which may have contained low viral copies. Previous study described that higher RSV viral load was associated with clinical severity^21^; however, RSV viral load data was not available in the current study. Furthermore, we did not take into account the co-infection with respiratory bacterial pathogens. In fact, it has been previously reported that RSV may increase the *Streptococcus pneumoniae* bacterial load which was associated with increased risk of radiologically-confirmed pneumonia^43^,^44^. It will be important to further investigate the underlying biological mechanism, interaction with nasopharyngeal bacteria that may leads to the clinical severity of RSV-A ON1.

## Conclusion

In conclusion, our current study highlights the clinical importance of RSV among the pediatric ARI cases in Central Vietnam. The emergence of RSV-A ON1 was associated with increased ARI hospitalization incidence. Furthermore, the ON1 ARI cases were associated with greater risk of LRTI, radiologically-confirmed pneumonia compared to the previously predominant NA1 genotype. Further molecular and clinical epidemiological studies on RSV-A ON1 genotype circulating across the world would be important for better understanding of RSV-A ON1’s clinical significance which may have impact on future vaccine development.

## Materials and Methods

### Study site and case enrollment

A population-based prospective pediatric ARI surveillance was established at Khanh Hoa province, Nha Trang, Central Vietnam in 2007. Khanh Hoa General Hospital (KHGH) is the provincial hospital and the only hospital in Nha Trang city. In Vietnam, all children less than 6 years of age are covered by a free government health insurance. Transportation system in Nha Trang city is considerably good as children living in the study area can reach KHGH within one hour. Therefore, access to medical care is relatively good in the study area. All children from the catchment area admitted to KHGH presenting with cough and/or difficulty breathing were recorded as ARI cases and enrolled in the current study. Written informed consents were obtained from the parents or guardians of the pediatric ARI cases to enroll in the study. Clinical-epidemiological information, chest radiographs result (Chest X-ray), laboratory test data and nasopharyngeal (NP) swab samples were collected from all the participants. The catchment area covered 198,729 individuals living in 42,770 households from 16 communities with 13,631 children less than 5 years of age. Detailed methods and characteristics of the study population have been described previously^4^.

### Ethics

This study was approved by the institutional ethical review boards of National Institute of Hygiene and Epidemiology (NIHE), Vietnam, and Institute of Tropical Medicine, Nagasaki University, Japan. The study was conducted in accordance with the approved guidelines.

### Study period

Pediatric ARI cases enrolled to the ARI surveillance in Nha Trang, Central Vietnam during the period of January 2010–December 2012 were selected and utilized for this study.

### Clinical data collection and categorization

Clinical categories were defined using modified World Health Organization (WHO) Integrated Management of Childhood Illnesses (IMCI) algorithms^45^. The presence of tachypnea (Respiratory Rate >60/min for children ≦ 1month, >50/min for 2–11 months and >40/min for 12–59 months) were categorized as mild LRTIs. Furthermore, children with general danger signs (situation in which children were either unable to drink, under convulsion or lethargy), chest-wall indrawing or stridor were categorized as severe LRTIs. Radiologically-confirmed pneumonia was defined as substantial alveolar consolidation or pleural effusion in chest X-ray result following the standardized interpretation method established by WHO Vaccine Trial Investigators Group^46^. Cases with abnormal shadow but not substantial alveolar consolidation or pleural effusion were considered as abnormal chest X-ray or other lower respiratory infection^46^.

### Virological investigation

Viral nucleic acids were extracted from patient’s NP swab samples using QIA viral RNA Minikit (QIAGEN Inc., Valencia, CA) following the manufacturer’s manual. Four Multiplex -PCR assays were performed for screening 13 respiratory viral pathogens including RSV, Influenza-A and B, Human Metapneumovirus, Parainfluenzavirus 1–4, Human Coronaviruses (229E, OC43), Adenovirus and Bocavirus. The detailed protocol of respiratory viruses screening was previously described^4^.

RSV-positive confirmed samples were further screened for subgroup (A/B) and genotype by amplifying and sequencing the 2nd hypervariable region of G-protein as previously described^1^,^47^. BigDye Terminator ver.3.1 (Applied Biosystem, Foster City, CA, USA) was utilized for the sequencing reaction, and nucleotide sequence analysis was performed with 3730 DNA Analyzer (Applied Biosystem, Foster City, CA, USA). Multiple nucleotide sequences were aligned and edited with ClustalW ver.1.8. Phylogenetic analysis was executed using the Neighbor-Joining method with bootstrap value of 1000 replicates for testing statistical significance of the tree topology using MEGA ver.5.2.2.

### Statistical analysis

For the categorical variables, either two-tailed Pearson Chi-squared or Fisher’s exact tests were performed to test the statistical difference in proportion between two independent groups. For the numerical variables, two-sample t-test was performed for mean value comparison, and Mann-Whitney U test was used for median comparison. In the multivariate analysis, Generalized Linear Model with log-binomial regression was applied to estimate adjusted Relative Risk (Adj RR) and 95% Confidence Interval (CI). To control demographic confounding variables in the multivariate regression analysis, both forward-selection step and biologically plausible approaches were taken into account. All the statistical analysis was performed using STATA ver.12.1 (StataCorp LP, College Station, TX, USA). *P*-values less than 0.05 were considered to be statistically significant.

## Additional Information

**How to cite this article**: Yoshihara, K. *et al.* Association of RSV-A ON1 genotype with Increased Pediatric Acute Lower Respiratory Tract Infection in Vietnam. *Sci. Rep.* **6**, 27856; doi: 10.1038/srep27856 (2016).

## Supplementary Material

Supplementary Information

## Figures and Tables

**Table 1:** Yearly incidence data for ARIs, LRTIs, RSV and RSV subgroups (A/B) in Nha Trang, Khanh Hoa province during January 2010–December 2012.

	Year (Jan 2010–Dec 2012)	January 2010–December 2010		January 2011–December 2011		January 2012–December 2012	
	Overall cases(n)	Number ofARI cases(n)	Incidence per 100,000population (95% CI)	Number ofARI cases(n)	Incidence per 100,000population (95% CI)	Number ofARI cases (n)	Incidence per 100,000population (95% CI)
Total enrolled ARI cases	(n = 1854)	n = 542	3976.2 (3654.4–4317.9)	n = 513	3763.5 (3450.2–4096.7)	n = 799	5861.6 (5473.1–6269.2)
Total LRTI cases	(n = 398)	n = 99	726.3 (590.7–883.5)	n = 53	388.8 (291.4–508.3)	n = 246	1804.7 (1587.9–2042.4)
RSV positive ARI cases	(n = 426)	n = 135	990.4 (831.0–1171.2)	n = 80	586.9 (465.6–729.9)	n = 211	1547.9 (1347.4–1769.5)
**RSV subgroup (A/B)**							
RSV-A single infection	(n = 253)	n = 63	462.2 (355.3–591.0)	n = 32	234.8 (160.6–331.3)	n = 158	1159.1 (986.3–1353.3)
RSV-B single infection	(n = 77)	n = 38	278.8 (197.4–382.4)	n = 25	183.4 (118.7–270.6)	n = 14	102.7 (56.2–172.3)
RSV-A and B co-detection	(n = 16)	n = 3	22.0 (4.5–64.3)	n = 2	14.7 (1.8–53.0)	n = 11	80.7 (40.3–144.4)

**Table 2:** Demographic and clinical characteristics comparison between RSV and Non-RSV pediatric ARI cases.

	Total pediatric ARI hospitalization cases during Jan 2010–Dec 2012 (n = 1854)		
	RSV positive ARI group (n = 426)	Non-RSV ARI group (n = 1428)	*p*-value#
	Total number (%)/Median (IQR¶)	Total number (%)/Median (IQR¶)	
Demographic information			
Male sex (%)	254 (59.6%)	799 (56.0%)	0.179
Median age (in month)	**12 (IQR: 5–21)**	17 (IQR: 9–29)	<0.001***
Age group (%)			
0–12month	218 (51.2%)	528 (37.0%)	<0.001***
13–24month	131 (30.8%)	442 (31.0%)	
25–36month	52 (12.2%)	187 (13.1%)	
37–48month	17 (4.0%)	94 (6.6%)	
49–60month	2 (0.5%)	(4.3%)	
>60month	6 (1.4%)	116 (8.1%)	
Socioeconomic status			
Daycare attendance (%)	137 (32.2%)	**552 (38.7%)**	0.015*
History			
Antibiotic used prior to hospitalization (%)	169 (39.7%)	**633 (44.3%)**	0.042*
Underlying medical condition (%)	133 (31.2%)	**541 (37.9%)**	0.012*
**Clinical information**	**Total number (%)/Mean (95% CI¶)**	**Total number (%)/Mean (95% CI¶)**	
Vital sign (s)			
Mean respiratory rate (per min)	**37.2 (95% CI: 36.3–38.1)**	35.7 (95% CI: 35.2–36.1)	0.003**
Mean body temperature (C.)	**37.9 (95% CI: 37.8–38.0)**	37.8 (95% CI: 37.8–37.9)	0.045*
SpO2 (≦90%)	12 (2.8%)	40 (2.8%)	0.986
Respiratory symptom and sign (s)			
Wheeze (%)	**225 (52.8%)**	655 (45.9%)	0.012*
Tachypnea (%)	**132 (31.0%)**	301 (21.1%)	<0.001***
Difficulty breathing (%)	51 (12.0%)	157 (11.0%)	0.575
Crackle (%)	**92 (21.6%)**	233 (16.3%)	0.012*
Cough (%)	426 (100%)	1423 (99.7%)	0.221
Chest-wall indrawing (%)	**42 (9.9%)**	97 (6.8%)	0.035*
LRTI and chest X-ray result			
LRTI† (%)	92 (21.6%)	307 (21.5%)	0.966
Mild LRTI (%)	43 (10.1%)	165 (11.6%)	0.402
Severe LRTI§ (%)	49 (11.5%)	142 (9.9%)	0.353
Abnormal chest X-ray (%)	62 (14.6%)	210 (14.7%)	0.856
Radiologically-confirmed pneumonia (%)	55 (12.9%)	182 (12.8%)	0.991
Treatment and outcome (s)			
Mean onset to hospitalization (in day)	2.4 (95% CI: 2.2–2.5)	2.4 (95% CI: 2.2–2.5)	0.831
Mean hospitalization duration (in day)	5.2 (95% CI: 4.9–5.4)	4.9 (95% CI: 4.7–5.1)	0.132
Antibiotic used (%)	426 (100%)	1428 (100%)	1.000
Steroid used (%)	213 (50.0%)	694 (48.6%)	0.612

^#^All the statistically significant *p*-values were indicated in bold font. As the index for the statistically significant values: * were used for *p*-value < 0.05, ** were for *p*-value < 0.01 and *** were for *p*-value ≦ 0.001.

^¶^IQR is abbreviation for Interquartile Range (1st and 3rd), and 95% CI is abbreviation for 95% Confidence Interval.

^†^LRTI is abbreviation for “lower respiratory tract infection” and based on the WHO definition of clinical pneumonia^45^.

^§^Severe lower respiratory tract infection (LRTI) was defined as presence of either danger sign, stridor or chest-wall indrawing.

**Table 3:** Yearly prevalence of RSV subgroups (A/B) and genotypes in Nha Trang, Khanh Hoa province during January 2010–December 2012.

	Pediatric ARI cases admitted to KHGH# (Jan 2010–Dec 2012)		
Year of sample collection	2010 (Jan–Dec)	2011 (Jan–Dec)	2012 (Jan–Dec)
Total ARI cases (n = 1854)	(n = 542)	(n = 513)	(n = 799)
**RSV positive ARI cases (n = 426)**	n = 135 (24.9%)	n = 80 (15.6%)	n = 211 (26.4%)
RSV subgroup A	63 (46.7%)	32 (40.0%)	158 (74.9%)
RSV subgroup B	38 (28.2%)	25 (31.3%)	14 (6.6%)
RSV-A and B mixed-infection	3 (2.2%)	2 (2.5%)	11 (5.2%)
***p*-value¶**	***p* = 0.711†**		***p* < 0.001§**
(Not classified)	31 (23.0%)	21 (26.3%)	28 (13.3%)
**RSV-A genotype (n = 269)**	(n = 66)	(n = 34)	(n = 169)
NA1 genotype	66 (100%)	34 (100%)	38 (22.5%)
ON1 genotype	0	0	123 (72.8%)
(Not classified)	0	0	8 (4.7%)
**RSV-B genotype (n = 93)**	(n = 41)	(n = 27)	(n = 25)
BA9 genotype	31 (75.6%)	17 (63.0%)	9 (36.0%)
BA10 genotype	6 (14.6%)	6 (22.2%)	0
BA-C genotype	1 (2.4%)	3 (11.1%)	8 (32.0%)
(Not classified)	3 (7.3%)	1 (3.7%)	8 (32.0%)
Total RSV-A&B genotype confirmed	n = 107 (in 2010)	n = 61 (in 2011)	n = 194 (in 2012)

^#^KHGH is abbreviation for Khanh Hoa General Hospital in Nha Trang city, Khanh Hoa province.

^¶^For the statistical tests for RSV subgroups (A/B) proportion comparison among sampling years, two-tailed Fisher’s exact tests were performed. Statistically significant p-value was indicated in bold font.

^†^p-value was for RSV subgroups (A/B) proportion comparison between year of 2010 and 2011.

^§^p-value was for RSV subgroups (A/B) proportion comparison among 2010, 2011 and 2012.

**Table 4:** Demographic and clinical characteristics comparison between RSV-A ON1 and NA1 genotype pediatric ARI cases.

	RSV subgroup A positive pediatric ARI cases during Jan 2010–Dec 2012 (n = 269)		
	RSV-A ON1 ARI group (n = 123)	RSV-A NA1 ARI group (n = 138)	*p*-value#
	Total number (%)/Median (IQR¶)	Total number (%)/Median (IQR¶)	
Demographic information			
Male sex (%)	76 (61.8%)	79 (57.3%)	0.456
Median age (in month)	**11 (IQR: 3–18)**	13 (IQR: 6–23)	0.020*
Age group (%)			
0–12month	68 (55.3%)	68 (49.3%)	0.198
13–24month	41 (33.3%)	40 (29.0%)	
25–36month	12 (9.8%)	22 (15.9%)	
37–48month	1 (0.8%)	6 (4.4%)	
49–60month	0	0	
> 60month	1 (0.8%)	2 (1.5%)	
Socioeconomic status			
Daycare attendance (%)	30 (24.4%)	54 (39.1%)	0.011*
History			
Antibiotic used prior to hospitalization (%)	55 (44.7%)	53 (38.4%)	0.832
Underlying medical condition (%)	53 (43.1%)	30 (21.7%)	< 0.001***
Respiratory virus co-infection (%)	27 (22.0%)	34 (24.6%)	0.609
**Clinical information**	**Total number (%)/Mean (95% CI¶)**	**Total number (%)/Mean (95% CI¶)**	
Vital sign (s)			
Mean respiratory rate (per min)	**38.8 (95% CI: 36.9–40.8)**	35.9 (95% CI: 34.4–37.5)	0.021*
Mean body temperature (C.)	38.0 (95% CI: 37.9–38.2)	37.9 (95% CI: 37.8–38.0)	0.261
SpO2 (≦90%)	**9 (7.3%)**	2 (1.5%)	0.028*
Respiratory symptom and sign (s)			
Wheeze (%)	**100 (81.3%)**	46 (33.3%)	<0.001***
Tachypnea (%)	**62 (50.4%)**	35 (25.4%)	<0.001***
Difficulty breathing (%)	**23 (18.7%)**	9 (6.5%)	0.004**
Crackle (%)	**46 (37.4%)**	25 (18.1%)	<0.001***
Cough (%)	123 (100%)	138 (100%)	1.000
Chest-wall indrawing (%)	19 (15.5%)	12 (8.7%)	0.092
LRTI and chest X-ray result			
LRTI†(%)	**42 (34.2%)**	21 (15.2%)	<0.001***
Mild LRTI (%)	**19 (15.5%)**	9 (6.5%)	0.027*
Severe LRTI§(%)	**23 (18.7%)**	12 (8.7%)	0.028*
Abnormal chest X-ray (%)	**28 (22.8%)**	13 (9.4%)	0.003**
Radiologically-confirmed pneumonia (%)	**24 (19.5%)**	12 (8.7%)	0.011*
Treatment and outcome (s)			
Mean onset to hospitalization (in day)	**1.7 (95% CI: 1.5–1.9)**	2.7 (95% CI: 2.3–3.0)	<0.001***
Mean hospitalization duration (in day)	5.3 (95% CI: 4.9–5.8)	5.1 (95% CI: 4.7–5.4)	0.329
Antibiotic used (%)	123 (100%)	138 (100%)	1.000
Steroid used (%)	69 (56.1%)	64 (46.4%)	0.117

^#^All the statistically significant *p*-values were indicated in bold font. As the index for the statistically significant values: * were used for *p*-value < 0.05, ** were for *p*-value < 0.01 and *** were for *p*-value ≦ 0.001.

^¶^IQR is abbreviation for Interquartile Range (1st and 3rd), and 95% CI is abbreviation for 95% Confidence Interval.

^†^LRTI is abbreviation for “lower respiratory tract infection” and based on the WHO definition of clinical pneumonia^45^.

^§^Severe lower respiratory tract infection (LRTI) was defined as presence of either danger sign, stridor or chest-wall indrawing.

**Table 5:** Multivariate log-binomial regression analysis of clinical severity comparison between RSV-A ON1 and NA1 genotype ARI cases.

Clinical manifestation (s)	RSV (subgroup/genotype)	Unadjusted RR	95% CI¶	Adjusted RR†	95% CI¶
Vital sign					
SpO2 (≦90%)	RSV-A ON1 ARI group (n = 123)	5.05	**1.11–22.92**	3.18	0.71–14.29
	RSV-A NA1 ARI group (n = 138)#	(ref.)	…	…	…
Clinical symptom and sign (s)					
Wheeze		**2.44**	**1.90–3.13**	**2.21**	**1.72–2.86**
Tachypnea		**1.99**	**1.42–2.78**	**1.83**	**1.30–2.57**
Difficulty breathing		**2.87**	**1.38–5.96**	**2.46**	**1.12–5.39**
Crackle		**2.06**	**1.35–3.15**	**1.96**	**1.28–3.00**
Chest-wall indrawing		1.78	0.90–3.51	1.95	0.88–4.31
LRTI and chest X-ray result					
LRTI§		**2.24**	**1.41–3.57**	**2.26**	**1.37–3.72**
Mild LRTI		**2.37**	**1.11–5.04**	1.94	0.90–4.19
Severe LRTIƒ		**2.15**	**1.12–4.14**	**2.42**	**1.12–5.25**
Abnormal chest X-ray		**2.42**	**1.32–4.46**	**2.14**	**1.13–4.04**
Radiologically-confirmed pneumonia		**2.25**	**1.18–4.30**	**1.98**	**1.01–3.87**

^#^In the log-binomial regression analysis, RSV-A NA1 ARI group (n = 138) was used as the reference group.

^¶^95% CI is abbreviation for 95% Confidence Interval.

^†^In the multivariate log-binomial regression, variables including sex, age, antibiotic use prior to hospitalization, daycare attendance, viral co-infection and underlying medical condition were adjusted for estimating adjusted Relative Risk (Adj RR) and 95% Confidence Interval (CI).

^§^LRTI is abbreviation for “lower respiratory tract infection” and based on the WHO definition of clinical pneumonia^45^.

^ƒ^Severe lower respiratory tract infection (LRTI) was defined as presence of either danger sign, stridor or chest-wall indrawing.

All the statistically significant values were indicated in bold font.
