# Supplementary material for: Association of RSV-A ON1 genotype with Increased Pediatric Acute Lower Respiratory Tract Infection in Vietnam
Source: Sci Rep. 2016 Jun 16;6:27856. doi: 10.1038/srep27856 (PMC4910061; doi:10.1038/srep27856)
Supplement: Supplementary Information [file srep27856-s1.pdf]

Title:

Association of RSV-A ON1 genotype with Increased Pediatric Acute Lower Respiratory Tract Infection in Vietnam

Authors list:

Keisuke Yoshihara[1,2], Minh Nhat Le[1,3], Michiko Okamoto[4], Wadagni Anita Carolle Akpeedje[1], Hien Anh Nguyen[3], Michiko Toizumi[1,5], Enga Pham[6], Motoi Suzuki[7], Ai Thi Thuy Nguyen[6], Hitoshi Oshitani[4], Koya Ariyoshi[5,7], Hiroyuki Moriuchi[5,8], Masahiro Hashizume[1,5], Duc Anh Dang[3], and Lay-Myint Yoshida[1,*]

Affiliations:

1. Department of Pediatric Infectious Diseases, Institute of Tropical Medicine, Nagasaki University, Nagasaki, 852-8523, Japan;
2. Leading Program, Graduate School of Biomedical Science, Nagasaki University, Nagasaki, 852-8523, Japan;
3. National Institute of Hygiene and Epidemiology, Hanoi, Vietnam;
4. Department of Virology, Tohoku University Graduate School of Medicine, Sendai, 980-8575, Japan;
5. Graduate School of Biomedical Sciences, Nagasaki University, Nagasaki, 852-8523, Japan;
6. Khanh Hoa General Hospital, Nha Trang, Vietnam;
7. Department of Clinical Medicine, Institute of Tropical Medicine, Nagasaki University, Nagasaki, 852-8523, Japan;
8. Department of Pediatrics, Nagasaki University Hospital, Nagasaki, 852-8102, Japan;

*Corresponding author: lmyoshi@nagasaki-u.ac.jp

Figure legend

Supplementary Figure 1. Phylogenetic analysis of RSV subgroup A G-protein 2nd hypervariable region circulating in Khanh Hoa province during January 2010 - December 2012

Supplementary Figure 2. Phylogenetic analysis of RSV subgroup B G-protein 2nd hypervariable region circulating in Khanh Hoa province during January 2010 - December 2012

Phylogenetic analysis was executed by the Neighbor-Joining method with bootstrap test of 1000 replicates using MEGA ver. 5.2.2 software. Bootstrap values higher than 70 were considered to be statistically significant and indicated at each branch node. Scale bar represents evolutionary distance calculated using the Maximum Composite Likelihood method. The length of scale bar corresponds to number of nucleotide substitutions per site from the closest branching node. Distinct colored circles were used as indicator for ARI samples originated from different sampling years (2010, 2011 or 2012): GREEN-filled circles were used for ARI samples obtained during Jan 2010 - Dec 2010, BLUE-filled circles for ARI samples during Jan 2011 - Dec 2011 and RED-filled circles for ARI samples during Jan 2012 - Dec 2012. “ARI- “is followed by four digits sample specific ID number. We intentionally excluded some ARI samples with identical nucleotide sequences particularly from same sampling year.

Supplementary Tables

Supplementary Table 1. Multivariate log-binomial regression analysis of clinical severity comparison between RSV and Non-RSV pediatric ARI cases

In the log-binomial regression analysis, RSV-positive ARI cases (n=426) and Non-RSV ARI cases (n=1428) detected during January 2010 - December 2012 were included. Non-RSV ARI cases (n=1428) were used as the reference group. In the multivariate analysis, demographic variables, including sex, age, antibiotic usage prior to hospitalization, daycare attendance, co-infection with other respiratory viruses and underlying medical condition were adjusted for estimating adjusted Relative Risk (Adj RR) and 95% Confidence Interval (CI).

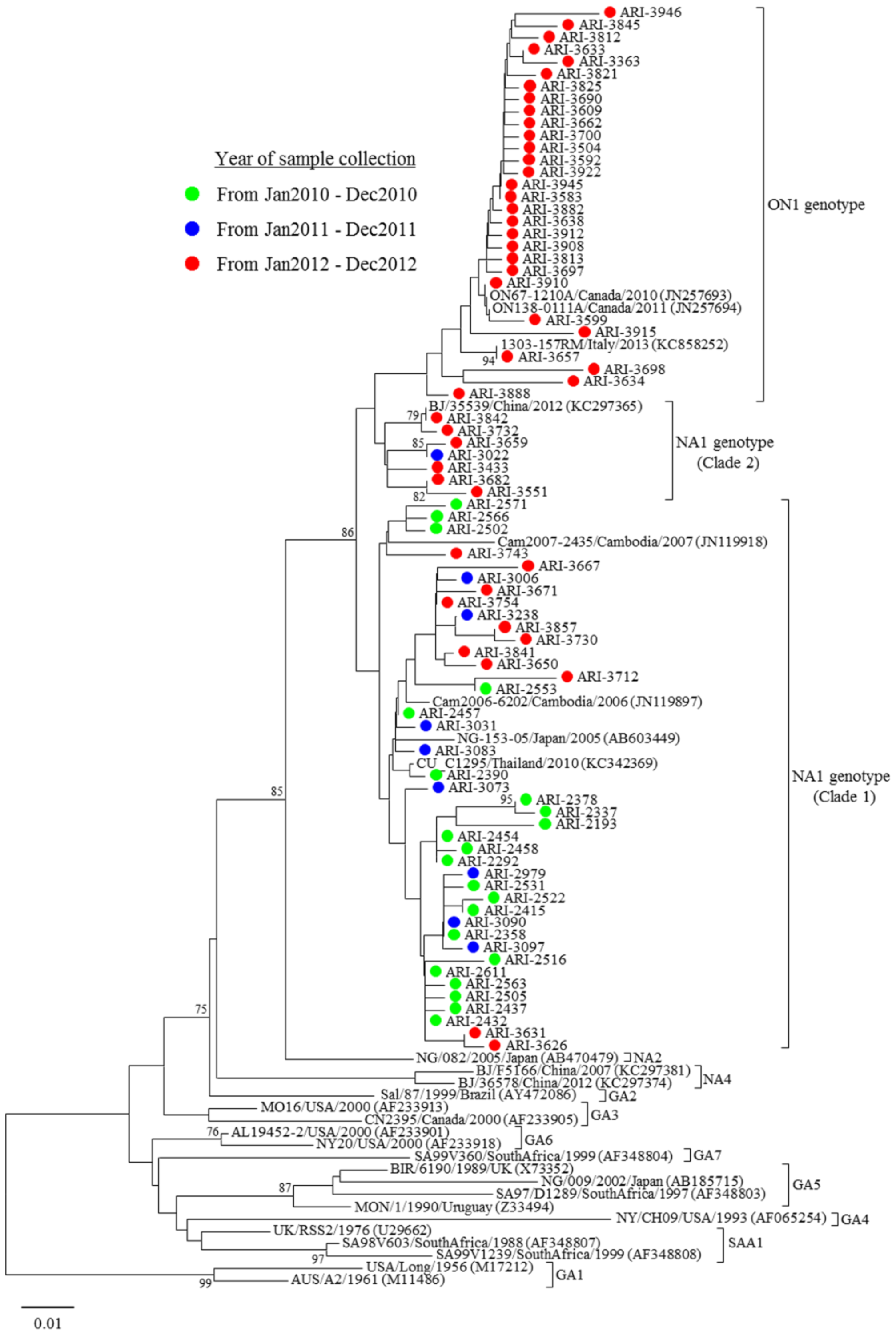
Year of sample collection
From Jan2010 - Dec2010
From Jan2011 - Dec2011
From Jan2012 - Dec2012
ARI-3946
ARI-3845
ARI-3812
ARI-3633
ARI-3363
ARI-3821
ARI-3825
ARI-3690
ARI-3609
ARI-3662
ARI-3700
ARI-3504
ARI-3592
ARI-3922
ARI-3945
ARI-3583
ARI-3882
ARI-3638
ARI-3912
ARI-3908
ARI-3813
ARI-3697
ARI-3910
ON67-1210A/Canada/2010 (JN257693)
ON138-0111A/Canada/2011 (JN257694)
ARI-3599
ARI-3915
1303-157RM/Italy/2013 (KC858252)
94
ARI-3657
ARI-3698
ARI-3634
ARI-3888
ON1 genotype
BJ/35539/China/2012 (KC297365)
79
ARI-3842
ARI-3732
ARI-3659
85
ARI-3022
ARI-3433
ARI-3682
ARI-3551
82
NA1 genotype
(Clade 2)
86
ARI-2571
ARI-2566
ARI-2502
Cam2007-2435/Cambodia/2007 (JN119918)
ARI-3743
ARI-3667
ARI-3006
ARI-3671
ARI-3754
ARI-3238
ARI-3857
ARI-3730
ARI-3841
ARI-3650
ARI-3712
ARI-2553
Cam2006-6202/Cambodia/2006 (JN119897)
ARI-2457
ARI-3031
NG-153-05/Japan/2005 (AB603449)
ARI-3083
CU_C1295/Thailand/2010 (KC342369)
ARI-2390
ARI-3073
85
95
ARI-2378
ARI-2337
ARI-2193
ARI-2454
ARI-2458
ARI-2292
ARI-2979
ARI-2531
ARI-2522
ARI-2415
ARI-3090
ARI-2358
ARI-3097
ARI-2516
ARI-2611
ARI-2563
ARI-2505
ARI-2437
ARI-2432
ARI-3631
ARI-3626
NA1 genotype
(Clade 1)
75
NG/082/2005/Japan (AB470479)
NA2
BJ/F5166/China/2007 (KC297381)
BJ/36578/China/2012 (KC297374)
NA4
Sal/87/1999/Brazil (AY472086)
GA2
MO16/USA/2000 (AF233913)
CN2395/Canada/2000 (AF233905)
GA3
76
AL19452-2/USA/2000 (AF233901)
NY20/USA/2000 (AF233918)
GA6
SA99V360/SouthAfrica/1999 (AF348804)
GA7
BIR/6190/1989/UK (X73352)
NG/009/2002/Japan (AB185715)
87
SA97/D1289/SouthAfrica/1997 (AF348803)
MON/1/1990/Uruguay (Z33494)
GA5
NY/CH09/USA/1993 (AF065254)
GA4
UK/RSS2/1976 (U29662)
SA98V603/SouthAfrica/1988 (AF348807)
97
SA99V1239/SouthAfrica/1999 (AF348808)
SAA1
USA/Long/1956 (M17212)
99
AUS/A2/1961 (M11486)
GA1
0.01

Fig. S1

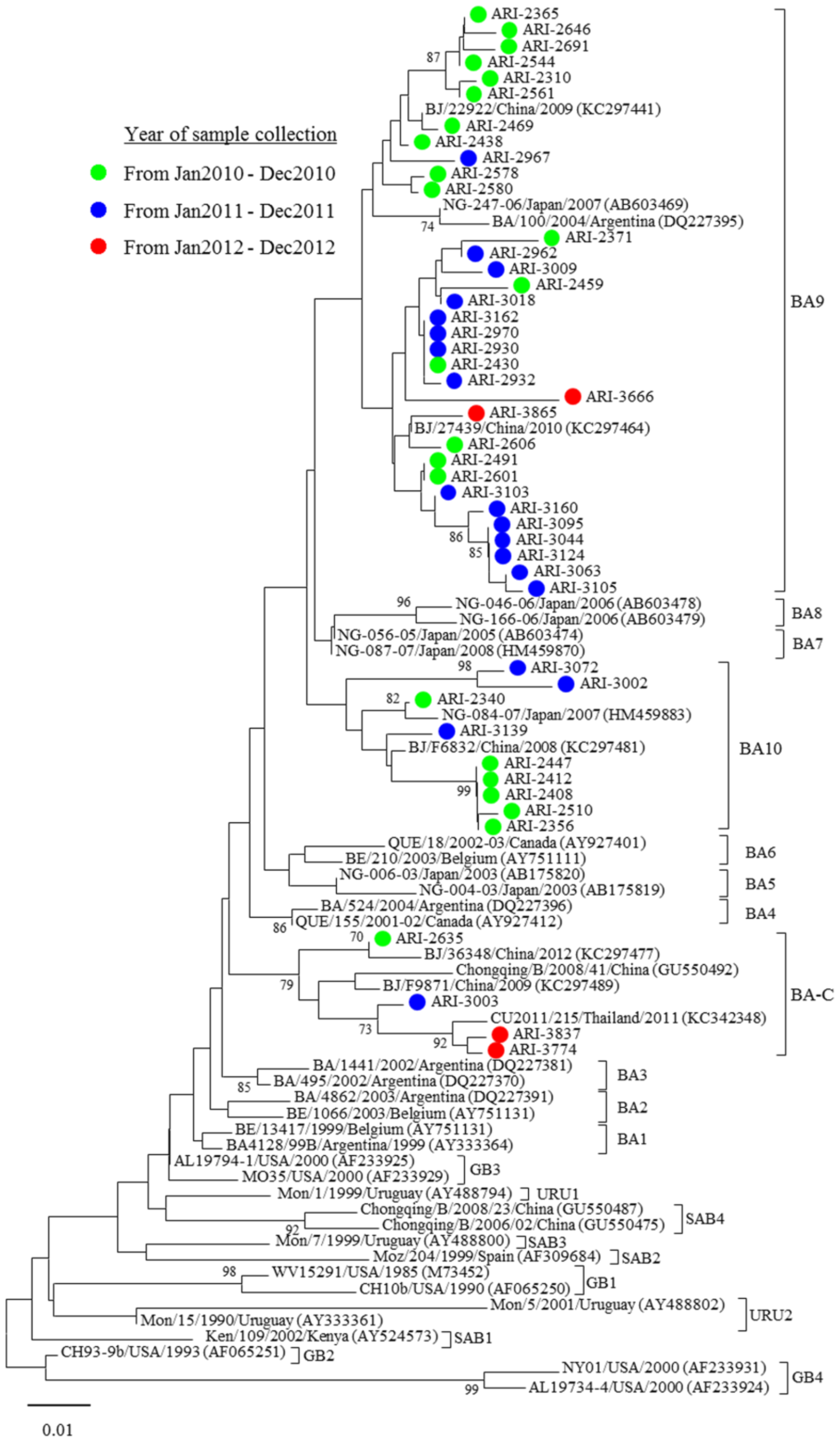

Year of sample collection
From Jan2010 - Dec2010
From Jan2011 - Dec2011
From Jan2012 - Dec2012
ARI-2365
ARI-2646
ARI-2691
87
ARI-2544
ARI-2310
ARI-2561
BJ/22922/China/2009 (KC297441)
ARI-2469
ARI-2438
ARI-2967
ARI-2578
ARI-2580
NG-247-06/Japan/2007 (AB603469)
74
BA/100/2004/Argentina (DQ227395)
ARI-2371
ARI-2962
ARI-3009
ARI-2459
ARI-3018
ARI-3162
ARI-2970
ARI-2930
ARI-2430
ARI-2932
ARI-3666
ARI-3865
BJ/27439/China/2010 (KC297464)
ARI-2606
ARI-2491
ARI-2601
ARI-3103
ARI-3160
ARI-3095
86
ARI-3044
85
ARI-3124
ARI-3063
ARI-3105
BA9
96
NG-046-06/Japan/2006 (AB603478)
NG-166-06/Japan/2006 (AB603479)
BA8
NG-056-05/Japan/2005 (AB603474)
NG-087-07/Japan/2008 (HM459870)
BA7
98
ARI-3072
ARI-3002
82
ARI-2340
NG-084-07/Japan/2007 (HM459883)
ARI-3139
BJ/F6832/China/2008 (KC297481)
BA10
ARI-2447
ARI-2412
99
ARI-2408
ARI-2510
ARI-2356
QUE/18/2002-03/Canada (AY927401)
BE/210/2003/Belgium (AY751111)
BA6
NG-006-03/Japan/2003 (AB175820)
NG-004-03/Japan/2003 (AB175819)
BA5
BA/524/2004/Argentina (DQ227396)
86
QUE/155/2001-02/Canada (AY927412)
BA4
70
ARI-2635
BJ/36348/China/2012 (KC297477)
Chongqing/B/2008/41/China (GU550492)
79
BJ/F9871/China/2009 (KC297489)
BA-C
ARI-3003
CU2011/215/Thailand/2011 (KC342348)
73
ARI-3837
92
ARI-3774
BA/1441/2002/Argentina (DQ227381)
85
BA/495/2002/Argentina (DQ227370)
BA3
BA/4862/2003/Argentina (DQ227391)
BE/1066/2003/Belgium (AY751131)
BA2
BE/13417/1999/Belgium (AY751131)
BA4128/99B/Argentina/1999 (AY333364)
BA1
AL19794-1/USA/2000 (AF233925)
MO35/USA/2000 (AF233929)
GB3
Mon/1/1999/Uruguay (AY488794)
URU1
Chongqing/B/2008/23/China (GU550487)
92
Chongqing/B/2006/02/China (GU550475)
SAB4
Mon/7/1999/Uruguay (AY488800)
SAB3
Moz/204/1999/Spain (AF309684)
SAB2
98
WV15291/USA/1985 (M73452)
CH10b/USA/1990 (AF065250)
GB1
Mon/5/2001/Uruguay (AY488802)
Mon/15/1990/Uruguay (AY333361)
URU2
Ken/109/2002/Kenya (AY524573)
SAB1
CH93-9b/USA/1993 (AF065251)
GB2
NY01/USA/2000 (AF233931)
99
AL19734-4/USA/2000 (AF233924)
GB4
0.01

Fig. S2

Supplementary Table 1. Multivariate log-binomial regression analysis of clinical severity comparison between RSV and Non-RSV pediatric ARI cases

Clinical manifestation (s)	RSV / Non-RSV ARI cases	Unadjusted RR	95% CI[¶]	Adjusted RR[†]	95% CI[¶]
Clinical symptom and sign (s)					
Wheeze	RSV positive ARI cases (n=426)	**1.15**	**1.04-1.28**	**1.16**	**1.03-1.30**
	Non-RSV ARI cases (n=1428)[#]	(ref.)	. . .	. . .	. . .
Tachypnea		**1.47**	**1.24-1.75**	**1.51**	**1.24-1.83**
			. . .	. . .	. . .
Crackle		**1.32**	**1.07-1.64**	**1.48**	**1.19-1.85**
			. . .	. . .	. . .
Chest wall indrawing		**1.45**	**1.03-2.05**	**1.60**	**1.05-2.44**
			. . .	. . .	. . .

[#] In the log-binomial regression analysis, Non-RSV ARI cases (n=1428) was used as the reference group.

[¶] 95%CI is abbreviation for 95% Confidence Interval.

[†] In the multivariate analysis using log-binomial regression, variables including sex, age, antibiotic use prior to hospitalization, daycare attendance, viral co-infection and underlying medical condition were adjusted for estimating adjusted Relative Risk (Adj RR) and 95% confidence Interval (CI).

All the statistically significant values were indicated in bold.
